# Global health education for medical students in Italy

**DOI:** 10.1186/s12909-021-02792-8

**Published:** 2021-06-24

**Authors:** Giulia Civitelli, Gianfranco Tarsitani, Veronica Censi, Alessandro Rinaldi, Maurizio Marceca

**Affiliations:** 1 Public Health and Infectious Diseases Department, Sapienza University of Rome, Piazzale Aldo Moro 5, 00185 Rome, Italy; 2 Italian Network for Global Health Education (INGHE), Rome, Italy; 3 Italian Society of Migration Medicine (SIMM – Società Italiana di Medicina delle Migrazioni), Rome, Italy; 4 Caritas Medical Area, Rome, Italy; 5 Italian Society of Medical Education (SIPEM – Società Italiana di Pedagogia Medica), Rome, Italy

**Keywords:** Global health, medical education, health equity, university’s third mission

## Abstract

**Background:**

Global health education (GHE) in Italy has spread since the first decade of 21st century. The presence of global health (GH) courses in Italy was monitored from 2007 to 2013. In 2019, a new survey was proposed to assess the availability of educational opportunities in Italian medical schools.

**Methods:**

An online survey was carried out using a questionnaire administered to a network of interested individuals with different roles in the academic world: students, professors, and members of the Italian Network for Global Health Education. The features of courses were analysed through a score.

**Results:**

A total of 61 responses were received from affiliates of 33 out of the 44 medical schools in Italy. The national mean of GH courses for each faculty was 1.2, reflecting an increase from 2007. The courses increased nationwide, resulting in a dispersed GHE presence in northern, central and southern Italy. One of the most critical points was related to the nature of “elective” courses, which were not mandatory in the curricula. Enrollees tended to be students genuinely interested in GH issues. Some community and service-learning experiences, referred to as GH gyms, were also detected at national and international levels.

**Conclusions:**

GHE has spreading in Italy in line with the vision of the Italian Network for Global Health Education. Although progress has been made to disperse GH courses around the country, more academic commitment is needed to include GH in the mandatory curricula of medical schools and other health faculties.

**Supplementary Information:**

The online version contains supplementary material available at 10.1186/s12909-021-02792-8.

## Background

In the last two decades Global Health Education (GHE) spread in in Europe and around the world, as an increasing number of scientific articles have shown [1–9]. Surveys on global health educational opportunities conducted in several countries offer insights into international developments in GHE early in the 21st century.

At the beginning of the century most medical schools were not well positioned to address the needs of global health training, despite both the strong, growing demand from medical students and the changing societal forces that call for it [10, 11]. In some countries, such as Sweden, there seemed to be a consensus that global health education could be a means to increase the fulfilment of knowledge and skills in topics such as socioeconomic determinants of health, global perspective on health-care systems, health promotion and disease prevention, human rights and ethics [12]. Some authors made the hypothesis that a substantial challenge facing the expansion of global health training within medical school curricula was the lack of consensus among schools about the necessary information and skills that need to be taught [13, 14].

In 2007, Rowson and colleagues carried out a survey of medical schools across the world in an effort to analyse the teaching of global health. Results indicated that the frequency of teaching GH was rising in prominence, particularly through global health elective/exchange programmes. Rowson et al., also found an increase in the teaching of subjects such as globalization and health, as well as international comparison of health systems. Their findings indicated that global health teaching was moving away from the previous focus on tropical medicine and towards issues of more global relevance [15].

In 2013, Khan and colleagues published a review of the state of GHE in US medical schools and elaborated recommendations for wider inclusion of GH in medical education [16]. They suggested the development of a basic curriculum applicable to all medical students, with progressively more advanced electives available. Based on the resources and interest of medical schools, they could choose to adopt a multi-level high-intensity model to target students with varying degrees of motivation, or a lower intensity model providing a baseline level of GH topics to all medical students, or a combination of both. In the case of global health electives abroad, a “best practice” relationship with a host institution was suggested.

A call for a more critical reflection on GH curricula, the evaluation of the desired profile of participants and the necessity of equity of access was made by Harmer and colleagues, who, in 2013, carried out a survey of undergraduate and postgraduate GHE programmes and courses in the U.K., and found that 15 universities offered at total of 25 postgraduate and six undergraduate GH degree programmes [17]. Similar conclusion were made by Kaffes and colleagues, who, in 2016, conducted an analysis of capacity, needs and barriers of GHE in Germany. The study demonstrated the need for a more systematic GHE in the country, which was impeded mainly by a lack of institutional priority and structure. The study recommended that GH educators engage in a debate on GH curricula with a focus on core competencies, an interdisciplinary approach and best teaching formats [18]. In Canada, in the same year, an analysis of the offerings of GHE underlined the presence of encouraging practices together with the existence of areas for improvement, which included the selection of students and alternative training formats [19]. Areas for improvement were also identified in India, where a 2017 survey showed fragmented delivery and a lack of focus on GHE at undergraduate and postgraduate levels, with the need to build greater interest among medical professionals [20].

Velden and colleagues gave a brief overview of GHE in the Netherlands in 2017. The research concluded that all eight university medical centres had incorporated GH aspects into their curricula and also offered specific GH courses. The authors underlined the importance of an internship in low- or middle-income countries in order to expose students to a foreign setting and situations of serious resource constraints [21]. In the U.K., Clarke and colleagues recently examined GH teaching in medical schools, and suggested “a global health Student Selected Component should be available to all students to provide the opportunity of further in-depth study for those who wish to advance their knowledge and skills in the area” [22]. In the core curriculum the authors suggested the inclusion of learning outcomes outlined in the General Medical Council’s 2018 “Outcomes for Graduates”, (e.g. p*opulation health; improvement and development of health; equity and sustainable healthcare; health service policy and economics; clinical guidelines; ecological, environmental and occupational hazards in ill-health and their mitigation*). Further global health topics that could be covered in the core curriculum were identified: t*ravellers’ and immigrant health; health and sustainable development; and health in relation to climate change.*

In 2017, Hau and colleagues published a systematic literature review of GH training among U.S. residency specialties. They reported wide disparities, with fewer opportunities among psychiatry and surgical residency specialties, and greater opportunities among medical residency specialties [23].

In Italy, GHE began to spread in the first years of 21st century, and has since received important stimulus from the European project “Equal opportunities for health”, coordinated by the NGO Doctors with Africa CUAMM^1^, with headquarters in Padua, Italy. Before this project, few Italian universities offered courses in this field. In 2010, the project led to the development of the Italian Network for Global Health Education (INGHE), a network of universities, scientific societies, non-governmental organisations and medical students’ associations interested in the promotion of GHE at undergraduate and postgraduate levels [24, 25]. INGHE’s members first agreed to a shared definition of global health (GH), then they defined the main objectives and contents of a GH course, the didactic methodologies that should be used, and the instruments for the evaluation of courses.^2^ At the end of 2014, INGHE decided to elaborate its reflections concerning GHE and medical education. A subsequent consensus process led to the development of the recently published paper ‘Medical education: an Italian contribution to the discussion on global health education’ [26]. In this paper it is explained why the spread of global health education in Italian universities is important. INGHE affirmed that GH is meant to be a new paradigm for health and health care, grounded in the theory of health determinants and it points out health inequities both within and among countries, framing them also through the lens of social justice. For this, according to INGHE teaching global health means introducing a new way to think and act concerning health while aiming to produce change in the community and in the whole society. This new approach to health should spread in the whole country, especially in front of the challenges related for example with inequities in health and health care, with the presence of migrants and refugees, and, recently, with the enormous consequences of Sars-Cov-2 pandemic in Italy, in Europe and in the whole world. It is increasingly important that future health professionals become able to face these challenges [27].

From 2007 to 2013, members of INGHE conducted four surveys to evaluate the availability of educational opportunities in Italian health faculties [28], followed by a pause in monitoring after the 2012–2013 academic year. Then, in 2019, INGHE proposed a new survey to assess the GH courses and educational opportunities offered in Italy in the 2018–2019 academic year. The research question was related to the number of GH courses in Italy and their characteristics. The authors’ hypothesis was that, since the last survey, there was an increase in the number of courses and improvement in their organization. The aim of the research is to assess the current situation of GHE so that it could be possible to compare the results with the previous surveys.

## Methods

The authors decided to readapt the questionnaire elaborated from the project “Equal opportunities for health”, so that it could be possible to compare the results with the previous surveys. The questionnaire was transformed into an online form and a section regarding GH gyms was added (see annexe). It investigated the main contents of courses, the didactic methodologies, the evaluation system, the availability of study material for students, the number of participants, the type of course (mandatory or elective), the possible number of university credits available, and the presence of GH gyms in the faculties. The survey was distributed through INGHE, by the Secretariat of the Italian Medical Students (SISM) - a student association present in all medical faculties in Italy and which is part of INGHE - and through the National Permanent Conference of the Presidents of Academic Degree Courses in Medicine. The survey was directed to members of INGHE, to representatives of medical students that are actively involved in global health and to representatives of academics who are part of the Conference (which involves the Presidents of all the Academic Degree in Medicine). The addresses were therefore in good positions to fill the questionnaire and provide meaningful answer.

The answers were collected between September 2019 and February 2020. The analysis was conducted in the following months.

A quantitative analysis of GH courses and their geographical distribution was made. The territorial subdivision proposed by the previous study (Bruno 2011), taken from the Italian National Institute of Statistics, was used to compare the results.

Two different scores were used to describe the courses: the first score (from 0 to 5) is the same used by Bruno (2011) and others; the second score (from 0 to 10) was created to describe the courses in a more detailed way. The two scores are described in Table 1. The criteria chosen for both scores derive from reflections made with the members of INGHE on GH courses, taking into account the medical education literature and adapting it to the academic Italian contest. According to INGHE, an ideal GH course should include at least one of the core arguments individuated by the network (social determinants of health, inequities in health, globalization and health) and interactive didactic methodologies; it should be longer than a single three hours lesson; university credits should be recognised to students; it should be open to students of different faculties, with a limited number of participants to allow more interactivity; it should be included in the mandatory curriculum; there should be an evaluation system and there should be the availability of study material.

**Table 1:** Description of scores used for courses evaluation

Score 1	Program	No program or no argument related with the INGHE definition	0	Score 2
		At least 1 argument of the INGHE definition	1	
	**Didactic Methodologies**	Lecture-style lessons	0	
		Interactive methodologies	1	
	**Length of the course**	Less than (or equal to) three hours	0	
		More than three hours	1	
	**University credits**	Without credits (no recognition from the faculty)	0	
		With credits	1	
	**Multiprofessional course**	Closed to other faculties	0	
		Open to other faculties	1	
	**Type of course**	Elective	0	
		Mandatory	1	
	**Presence of a referring person inside university**	No	0	
		Yes	1	
	**Presence of a pre-set limited number of participants**	No	0	
		Yes, more than or equal to 80 people^1^	0.5	
		Yes, less than 80 people	1	
	**Evaluation system**	None	0	
		Evaluation of appreciation OR of knowledge	0.5	
		Evaluation of appreciation AND of knowledge	1	
	**Availability of study material**	No	0	
		Yes	1	

^1^Eighty is the average number of participants in the mapped courses.

With the first score, the courses were divided in low level (≤ 1); medium level (2–3); high level (≥ 4).

With the second score, the courses have been divided in low level (≤ 3), medium-low level (4-5.5), medium-high level (6-7.5) and high level (≥ 8).

## Results

A total of 61 answered questionnaires were returned in the period from September 2019 to February 2020: 20 responses were from students, 22 from members of the nongovernmental organization Doctors with Africa CUAMM, 17 from people who work within universities, two from another nongovernmental organization, and one from a non-medical faculty member. From a total of 44 medical schools present in Italy, 33 (75 %) were reached through the questionnaire.^3^For 9 medical school, more than one answer has been given. The multiple answers for a single medical school, given by different people, have been analysed to understand if they referred to different courses or to the same educational proposal. A total of 38 GH courses were individuated, of which four were made from May to June 2018, thus they relate to the previous academic year. Seven of the 38 courses (18.4 %) belonged to formal programs of mandatory courses. Thirty-one courses (81.6 %) were elective, with a medium length of 9.5 h (range from 2 to 24 h). Concerning geographical distribution, 9 (23.7 %) courses were in the south, 9 (23.7 %) in the centre and 20 (52.6 %) in the north of Italy.

Of the 38 courses, 35 (92 %) were offered to medical students, of which 26 (68 %) were offered exclusively to medical students and 9 (24 %) were open also to other degrees or faculties. The other three courses, all in northern Italy, were offered to students of economy, sociology and nursing.

The national mean of GH courses offered over the total number of medical schools in Italy is 0.79 (with 0.89 in the north, 0.81 in the central region, 0,64 in the south). The national mean of GH courses over the medical schools reached through the questionnaire was 1.1 with a standard deviation of 0.87 (see Table 2).

**Table 2:** Mean and standard deviation of GH courses in Italy for medical schools reached through the questionnaire, divided for geographical distribution

	North	Centre	South	National
2007–2010	1.24 (1.53)	0.57 (0.51)	0.35 (0.79)	0.79 (1.20)
2009–2010	1.81 (1.38)	0.87 (0.35)	0.33 (0.65)	1.11 (1.18)
2018–2019	1.25 (0.76)	1.1 (0,54)	0.75 (0.84)	1.1 (0.87)
	*(20 courses over 16 medical schools reached)*	*(9 courses over 8 medical schools reached)*	*(9 courses over 12 medical schools reached)*	*(38 courses over 33 medical schools reached)*

Ten courses had a limited number of participants with a range from 15 to 250, and the average number of participants was 83. With regard to didactic methodologies, more than 55 % of courses had a percentage of lecture-style instruction higher than 50 %.

Ten courses included an evaluation of participants’ knowledge (four in mandatory courses and six in elective courses), and 12 courses made an evaluation of appreciation.

According to Score 1 (see Table 1), the distribution of courses in the different region is shown in Table 3.

**Table 3:** Distribution of courses according to Score 1

	LOW	MEDIUM	HIGH	TOTAL
**South**	0	2 (22.2 %)	**7 (77.7 %)**	9
**Centre**	0	**5 (55.5 %)**	4 (44.4 %)	9
**North**	0	**11 (55 %)**	9 (45 %)	20
**Italy**	0	18 (47.4 %)	20 (52.6 %)	38

According to Score 2, the distribution of courses in the different region is shown in Table 4.

**Table 4:** Distribution of courses according to Score 2

	LOW (≤ 3)	MEDIUM LOW (4-5.5)	MEDIUM HIGH (6-7.5)	HIGH (≥ 8)	TOTAL
**South**	3 (33.3 %)	**4 (44.4 %)**	2 (22.2 %)	0	9
**Centre**	1 (11.1 %)	**4 (44.4 %)**	3 (33.3 %)	1 (11.1 %)	9
**North**	**9 (45 %)**	2 (10 %)	4 (20 %)	5 (25 %)	20
**Italy**	**13 (34.2 %)**	10 (26.3 %)	9 (23.7 %)	6 (15.8 %)	38

For every scored category listed in Table 1, the authors analysed the mean value for the 38 GH courses.

As stated in the method section, the criteria of analysis have been chosen from the characteristics of an ideal GH course individuated by INGHE.

From this analysis, it emerged that positive points are the presence of at least one core arguments of GH (as individuated by INGHE) in all courses, the widespread presence of interactive didactic methodologies, and the length of courses (more than a single three hours lesson). Half of the 38 GH courses had a referring person inside the university and the 55 % of courses recognised university credits to participants.

The most critiqued points were the limited access for multiprofessionals (mean value of 0.26, where the majority of courses were offered only to medical students), the type of course (mean value of 0.18, where the majority of courses were elective), the pre-set limited number (mean value of 0.2, where the majority of courses had a number of participants higher than 80) and evaluation system (mean value of 0.29, where the majority of courses did not have an evaluation system).

In connection with GH gyms, 18 answers referred to national and international experiences. GH gyms at the national level included:

- Internship of medical students in Caritas Medical Centre, Rome [29].
- Internship of medical students and students of other health professions in the field of “health in prison” in Rome.
- Internship of medical students and students of other health professions in the reality if asylum seekers and refugees.
- Internship of medical students in prison in Milan.
- Internship in medical centre for homeless in Varese.
- A national course on global health organized by medical students for other medical students “Laboratorio di mondialità” (World Health Laboratory).

GH gyms at the international level included:

- Experiences for medical students organized by “Doctors with Africa CUAMM” in collaboration with the Secretariat of the Italian Medical Students (SISM) in Wolisso, Ethiopia and Tosamaganga, Tanzania.
- Experiences for residents offered by “Doctors with Africa CUAMM”.
- International experiences offered in Milan and Varese in collaboration with Non-Governmental associations.

## Discussions

After some years a new survey of GH courses and educational experiences was necessary to assess the situation of GHE in Italy. The results of the survey show that GH courses are present in different universities in Italy, and they have increased from a mean of 0.79 courses per medical school from 2007 to 2010 to 1.1 courses per medical school in the 2018–2019 academic year. Courses have recently spread throughout the country and there has been an important reduction in the gap in GH course distribution between northern, central and southern regions of Italy. Another important aspect of GHE in Italy, which is coherent with the vision of GH given by INGHE, was the involvement of students and of non-governmental organizations (especially Doctors with Africa CUAMM) in the organization of courses. The bottom-up approach was a specific characteristic of the work of INGHE and the authors believe it is a very important way to continue the spread of GHE.

As already stated in the results section analysing the scores, there are both positive and critique points of GH courses individuated through the questionnaire. Work should be done especially on the type of courses, on the inclusion of students from different faculties, on the evaluation system and the number of participants (a pre-set limited number of participants could facilitate learning with interactive didactic methodologies).

Another important point concerns the GH gym: the survey shows the presence of different types of offers for students who want to make an experience in the field, both at national or international levels. The main shortcoming of GH gyms was related to the fact that those experiences were limited to only a few medical schools, except those offered by Doctors with Africa CUAMM, which had the potential to reach all medical students.

The authors are aware that important limitations of the study relate to the lack of information for 11 Italian medical schools and the limited responses from university staff (72 % of responses were from students and NGOs). The limitation related with who answered the questionnaire on one side could be a consequence of the methodology used for the spread of the survey, on the other side it could be interpreted as an indicator that some academic professionals who were invited to participate were not committed to furthering the understanding of GHE. A possible future development of the research could be to compare those data with publicly available courses/ programs information, courses documentation, medical course accreditation reports and targeted interview data with academics from each medical program in Italy.

Nevertheless, the authors believe that this survey could be a good re-starting point to evaluate the offer of global health education in the country. Some limits of Italian GHE experiences emerge from the analysis of the two scores, especially from Score 2, which shows that a commitment is needed to improve the offer of GHE. The most problematic aspect is related with the type of courses: GH courses are mainly electives and are not recognised as mandatory. Other problems include the frequent absence of an evaluation system, and the lack of access to GHE by multiprofessionals since the courses are often offered only to medical students.

## Conclusions

GHE has continued to spread in Italy since 2007. The number of GH courses has increased throughout the country, coherently with the vision of INGHE. Some work has been done, but more work is needed to insert GH education in the mandatory curricula of medical schools. This article helps to quantify the current situation of GHE at the national level in Italy. The authors recognise the importance of GHE and propose to insert the main themes related with GH (social determinants of health, inequities in health, globalization and health, migration and health, international health cooperation) in the mandatory curricula of medical schools. The relevance of GHE is clearer than ever, especially considering the challenges related to the COVID-19 pandemic, during which inequities in health between different populations are once again evident and demonstrated [30–34]. The authors hope that this survey, together with other articles which are already published or which forthcoming, could give an important contribution to the debate on GHE in Italy.

## Supplementary information

**Additional file 1.**

## Data Availability

The datasets used and/or analysed during the current study are available from the corresponding author on reasonable request.

## Abbreviations

GH: global health
GHE: global health education
INGHE: Italian Network for Global Health Education

## Footnotes

1 Founded in 1950, Doctors with Africa CUAMM was the first non-governmental organization focused on healthcare to be recognized by the Italian government. It is now the country’s leading organization working to protect and improve the wellbeing and health of vulnerable communities in Sub-Saharan Africa. CUAMM also carries out capacity-building activities and conducts and disseminates scientific research with the end goal of ensuring that the fundamental human right to health can be enjoyed by everyone everywhere. More information on the website https://doctorswithafrica.org/en/

2 Documents produced and activities promoted by INGHE are available online at http://educationglobalhealth.eu/en/

3 According to official documents of the Ministry of Education, University and Research, in 2018 there were 44 medical schools in Italy, divided in this way: 14 in the south, 11 in the centre and 19 in the north of Italy.
