# Supplementary material for: Global health education for medical students in Italy
Source: BMC Med Educ. 2021 Jun 24;21:355. doi: 10.1186/s12909-021-02792-8 (PMC8222702; doi:10.1186/s12909-021-02792-8)
Supplement: Supplementary file 1 — Additional file 1. [file 12909_2021_2792_MOESM1_ESM.docx]

**GLOBAL HEALTH EDUCATION FOR MEDICAL STUDENTS IN ITALY**

*Mandatory questions

**INTRODUCTION**

1. University* ____________________
2. Faculty* ______________________
3. Surname and name of informant* __________________________
4. Role of the informant
   - Student
   - Resident
   - PhD student
   - Researcher
   - Professor
   - Someone else_________________
5. Was there a global health course in your university in the 2018/2019 academic year? *
   - Yes (go to question 6)
   - No (go to question 34)
   - I do not know (go to question 34)

**CHARACTERISTIC OF THE COURSE**

1. Title of the course* ___________________________________
2. Surname and name of the person in charge of the course* ___________________________
3. Role of the person in charge of the course* ____________________________
4. Phone number (if possible) ____________________________
5. Email address* ______________________________
6. Please write the surname, name, role and email address of person(s) who collaborate in the organization of the course. ________________________________________________________
7. To which degree course/courses is the course directed? *:
   - Medicine and Surgery
   - Nursing
   - Obstetrics
   - Rehabilitation sciences
   - Pharmacy
   - Social Service
   - Economy
   - Anthropology
   - Sociology
   - Other courses: ________________________________________________-
8. Type of educational activity*:
   - Mandatory
   - Elective
   - Something else: _____________-
9. Number of hours of teaching*: ______________
10. Number of university educational credits*: _______________
11. Academic year of the initial course*: __________________
12. Number of courses since the initial course began: __________________
13. Is there a limit to the number of participants in the course?
    - Yes
    - No
14. If you answered Yes to the question 18, what is the maximum number of participants? _________
15. Number of participants in the latest course: ____________________
16. In which year of their degree course could students take part in the global health course? ___________

**ORGANIZATION OF THE COURSE**

1. Didactic methodologies*
   - Lecture style lessons
   - Plenary discussion
   - Working groups
   - Case study
   - Role playing
   - Peer education
   - Narrative approach
   - Other: _________________________
2. What percentage of time is dedicated to the lecture-style lessons? *
   - Less than 25%
   - From 25% to 50%
   - From 50% to 75%
   - More than 75%
3. Which are the main issues of the course? *
   - Social determinants of health
   - Inequities in health and health care
   - Globalization and health
   - Origin and development of health systems
   - Migration and health
   - International health cooperation
   - Other: _____________________________________-
4. Please write the surname, name, role and email address of professors involved in the course. _______________________________

**EVALUATION OF THE COURSE**

1. Is there an evaluation of knowledges of participants? *
   - Yes
   - No
2. If there is an evaluation of knowledges, this is realized through:
   - Pre-test
   - Post-test
   - Short thesis
   - Oral exam
   - Other: _______________________
3. Is there an evaluation of the appreciation of participants?
   - Yes, at the end of every lesson
   - Yes, at the end of the course
   - No
4. Are there other types of evaluation? ____________________________
5. Is the didactic material available?
   - Yes
   - No
6. If you answered Yes to the question 30, the didactic material is:
   - On-line
   - On paper
   - Other: ___________________________________
7. The didactic material includes:
   - The on-line Global Health course available on the website of “Doctors with Africa CUAMM”
   - The website [www.saluteinternazionale.info](http://www.saluteinternazionale.info)
8. Is the person in charge of the course going to give the course the next academic year?
   - Yes
   - No
   - I do not know

**GLOBAL HEALTH GYM**

1. Do you know of global health educational experiences offered to medical students or to students of other health profession degree courses, which take place in the field? (For example, in the context of migration and health, health in prison, or international health cooperation.) *
   - Yes
   - No
2. If you answered Yes to the question 34, what is the title of the educational experience?

________________________________________________________________

1. In which context does the educational experience begin? _______________________________________
2. To whom is directed? (Students of which degree course?) ___________________________________
3. Length of the experience: _______________________________
4. Who is involved in the educational experience? Who will students meet during the experience? ___________________________________________________________
5. Is the experience officially recognised by the university? ____________________________
6. Is there an evaluation of the educational experience?
   - Yes
   - No
7. If you answered Yes to the question 42, what kind of evaluation is proposed to participants? _____________________
8. What are the results of the evaluation? ___________________________________________
9. Please write the surname, name, role and email address of person(s) in charge of the educational experience. _______________________________________________________________________
